# Formation and Stabilization of W_1_/O/W_2_ Emulsions with Gelled Lipid Phases

**DOI:** 10.3390/molecules26020312

**Published:** 2021-01-09

**Authors:** Anna Molet-Rodríguez, Olga Martín-Belloso, Laura Salvia-Trujillo

**Affiliations:** Department of Food Technology, University of Lleida—Agrotecnio Center, Av. Alcalde Rovira Roure 191, 25198 Lleida, Spain; anna.molet@udl.cat (A.M.-R.); olga.martin@udl.cat (O.M.-B.)

**Keywords:** W_1_/O/W_2_ emulsions, lipid phase gelation, chlorophyllin, clarification, encapsulation efficiently, storage conditions

## Abstract

Water-in-oil-in-water (W_1_/O/W_2_) emulsions are emulsion-based systems where the dispersed phase is an emulsion itself, offering great potential for the encapsulation of hydrophilic bioactive compounds. However, their formation and stabilization is still a challenge mainly due to water migration, which could be reduced by lipid phase gelation. This study aimed to assess the impact of lipid phase state being liquid or gelled using glyceryl stearate (GS) at 1% (*w*/*w*) as well as the hydrophilic emulsifier (T80: Tween 80 or lecithin) and the oil type (MCT:medium chain triglyceride or corn oil (CO) as long chain triglyceride) on the formation and stabilization of chlorophyllin W_1_/O/W_2_ emulsions. Their colloidal stability against temperature and light exposure conditions was evaluated. Gelling both lipid phases (MCT and CO) rendered smaller W_1_ droplets during the first emulsification step, followed by formation of W_1_/O/W_2_ emulsions with smaller W_1_/O droplet size and more stable against clarification. The stability of W_1_/O/W_2_ emulsions was sensitive to a temperature increase, which might be related to the lower gelling degree of the lipid phase at higher temperatures. This study provides valuable insight for the formation and stabilization of W_1_/O/W_2_ emulsions with gelled lipid phases as delivery systems of hydrophilic bioactive compounds under common food storage conditions.

## 1. Introduction

Water-in-oil-in-water (W_1_/O/W_2_) emulsions consist of a water-in-oil (W_1_/O) emulsion dispersed in an outer aqueous phase. The most common method to fabricate W_1_/O/W_2_ emulsions consist of a two-step process in which an initial step of making the W_1_/O emulsion is followed by its dispersion in another aqueous phase [1]. Due to the compartmentalized internal structure, W_1_/O/W_2_ emulsions offer great potential for the encapsulation and controlled release of hydrophilic bioactive compounds [2,3]. However, in these systems, multiple instability mechanisms can occur during their production and storage, such as coalescence of the lipid droplets and/or inner water droplets, and the coalescence of the inner water droplets with the outer water phase, which leads to water migration between both water phases. Also, inner water droplets may shrink or swell as a result of the water transfer between the inner and the outer water phases [4]. In addition, it is well known that the temperature and light during storage can also affect the stability of emulsion-based systems [5,6,7]. However, there is still scarce scientific evidence about the impact of the storage conditions on the stability of W_1_/O/W_2_ emulsions [8]. In principle, in order to form a W_1_/O/W_2_ emulsion, an initial lipophilic emulsifier is used for the stabilization of the inner interface. Polyglycerol ester of polyricinoleic acid (PGPR) has been demonstrated to be highly effective as W_1_/O stabilizer, due to its ability to form small W_1_ droplets, and because it forms a physical barrier around these droplets preventing their coalescence [3,9]. To stabilize the second interface, it is necessary to use a hydrophilic emulsifier. In this case, it has been reported that polysorbates, lecithin and proteins can be effective stabilizers of this secondary interface [4,10,11]. In W_1_/O/W_2_ emulsions, the lipid phase is filled with nanometric inner water droplets, implying that the size of the lipid droplets is typically larger than 1 µm, which in turn renders highly unstable emulsions [12]. Therefore, strategies for improving the colloidal stability of W_1_/O/W_2_ emulsions are necessary, which require modifying the properties of both the lipid and aqueous phases. On the one hand, through adding biopolymers in the outer aqueous phase to increase its viscosity, the oil droplet mobility can be reduced and subsequently the colloidal stability of W_1_/O/W_2_ emulsions may be enhanced [4]. On the other hand, increasing the viscosity of the lipid phase by formulating solid, semi-solid or gelled lipid phases, as well as decreasing its polarity, may minimize the diffusion rates between the two aqueous phases avoiding the destabilization of the inner water phase both during the emulsion formation and during their storage [8,13]. For instance, glyceryl monostearate (GS), a monoacylglycerol mainly composed of stearic acid (C18:0), has been recently used as an ingredient to gel lipid phases of O/W nanoemulsions through the formation of a crystal network [14,15]. However, to the best of our knowledge, the use of GS to formulate gelled lipid phases in order to improve the colloidal stability of W_1_/O/W_2_ emulsions has not been studied.

Therefore, the aim of the present work was to study the impact of the lipid phase composition and lipid phase state (liquid vs. gelled) on the formation and stabilization of W_1_/O/W_2_ emulsions. Specifically, they were formulated with two oil types with different triglyceride chain lengths, being medium chain triglyceride (MCT) and a long chain triglyceride oil being corn oil (CO). In addition, the formulation of gelled lipid phases of both oils was conducted by adding 1% (*w*/*w*) of GS in the lipid phase. Moreover, the use of different hydrophilic emulsifiers (Tween 80, T80; or lecithin) was also studied for the formation of W_1_/O/W_2_ emulsions. Droplet size and microscopy characterization of the inner W_1_ droplets and the W_1_/O droplets was measured. The stability of the formulated W_1_/O/W_2_ emulsions against storage under different environmental stresses, namely temperature (4, 25 or 35 °C) or light exposure, were also evaluated. Finally, chlorophyllin (CHL) was used as model hydrophilic compound for the evaluation of the formulated W_1_/O/W_2_ emulsions as delivery systems. Encapsulation efficiency (EE) immediately after the formulation of W_1_/O/W_2_ emulsions and during storage was determined.

## 2. Materials and Methods

### 2.1. Materials

MCT (Myglyol, Oxi-med expres) (99.9% of purity) and CO (Koipesol Asua, Deoleo, Spain) were used as a lipid phase. Sunflower oil, which was kindly donated by Borges (Lleida, Spain), was the dispersant in droplet size measurements. GS (Imwitor^®^ 491) with a purity of 96.7% (0.8% free glycerol and 95.9% monoglycerides) was used to formulate the gelled lipid phases. PGPR from castor oil (Grinsted^®^, DuPont Danisco NHIB Iberica S. L, Barcelona, Spain) was utilized as lipophilic emulsifier. Tween 80 (Lab Scharlab, Barcelona, Spain) and L-α-soybean lecithin, acquired from Alfa Aesar (Thermo Fisher Scientific, GmbH, Karlsruhe, Germany), were used as food-grade hydrophilic emulsifiers. CHL (coppered trisodium salt) with a molecular weight of 724.15 g/mol, copper contain of 3.5–6.5% and a purity of ≥95% was purchased from Alfa Aesar (Thermo Fisher Scientific, GmbH, Karlsruhe, Germany). Sodium alginate (MANUCOL^®^DH) was obtained from FMC Biopolymer Ltd. (Scotland, UK). NaCl POCH S.A. (Gliwice, Poland) was added to both of the inner and outer aqueous phases of the system, in order to adjust the osmotic pressure balance between the aqueous phases. Ultrapure Milli-Q water obtained from a Millipore filtration system (Merck, Darmstadt, Germany) was used for the preparation of all W_1_/O/W_2_ and solutions.

### 2.2. Formation of W_1_/O/W_2_ Emulsions 

W_1_/O/W_2_ were prepared according to a two-step emulsification method previously used by Artiga-Artigas et al. [3] with some modifications, consisting on the formation of the W_1_/O emulsion followed by its dispersion in an outer aqueous phase.

#### 2.2.1. Formation of the W_1_/O Emulsions

W_1_/O emulsions with liquid and gelled lipid phases were formulated using pure MCT or CO as the lipid phase and GS (1% *w*/*w*) as oleogelator. The lipid phase of the W_1_/O emulsions was prepared by dissolving PGPR in the lipid phase (MCT or CO) using a magnetic stirrer (450 rpm) for 5 min at 50 °C. To form the gelled lipid W_1_/O emulsions, GS was firstly melted by increasing the temperature at 50 °C and subsequently mixed with each of the previously prepared lipid phase containing PGPR. The visual appearance of the lipid phases being liquid or gelled at 4, 25 or 35 °C is presented in Table 1. Emulsion were formed by mixing the lipid phase with an aqueous phase consisting on 112.5 ppm CHL, 0.05 M NaCl and 2% (*w*/*w*) sodium alginate by using a laboratory T25 digital Ultra-Turrax mixer (IKA, Staufen, Germany) working at 11,000 rpm for 5 min. The temperature during the first emulsification step was kept at 50 °C in order to maintain the lipid phase in liquid state. Once the W_1_/O emulsions were formed, their temperature was reduced down to 4 °C for 2 h in order to allow lipid gelation in those emulsions with GS.

#### 2.2.2. Formation of the W_1_/O/W_2_ Emulsions

The second step was the dispersion of the previously prepared W_1_/O emulsions in the outer aqueous phase using a laboratory T25 digital Ultra-Turrax mixer (IKA, Staufen, Germany) working at 4000 rpm for 2 min. From each W_1_/O emulsion, two different W_1_/O/W_2_ emulsions were formed. On the one hand, the W_1_/O emulsion was dispersed in an outer aqueous phase containing NaCl 0.05 M, sodium alginate (2% *w*/*w*) and T80 (2% *w*/*w*). On the other hand, a percentage of 2% *w*/*w* of lecithin was previously mixed with the W_1_/O emulsion (18% *w*/*w*) using a magnetic stirrer at 750 rpm during 5 min followed by its dispersion in an outer aqueous phase containing NaCl 0.05 M and sodium alginate (2% *w*/*w*).

### 2.3. Initial Characterization of W_1_/O Emulsions and W_1_/O/W_2_ Emulsions

#### 2.3.1. Droplet Size

The mean droplet diameter (nm) of the W_1_/O emulsions were measured by dynamic light scattering (DLS) using a Zetasizer Nano-ZS laser diffractometer (Malvern Instruments Ltd., Worcestershire, UK) working at 633 nm and 25 °C, equipped with a back scatter detector (173°). Refractive indexes (RIs) of MCT and CO were 1.48 and 1.47, respectively. Samples were prior diluted in hexane, whose RI was 1.38, using a dilution factor of 1:9 sample-to-solvent. The inner W_1_ droplet size was characterized by average droplet size (nm). The droplet size of W_1_/O/W_2_ emulsions was measured by the static light scattering (SLS) technique with a Mastersizer 3000™ (Malvern Instruments Ltd., Worcestershire, UK). Samples were dispersed in distilled water (RI = 1.33) at 2200 rpm and the oil droplet size was reported as volume-weighted average (D_[4;3]_).

#### 2.3.2. Optical Microscopy Analysis

Phase contrast microscopy images of the W_1_/O/W_2_ emulsions were taken with an optical microscope (BX41, Olympus, Göttingen, Germany) using a ×100 oil immersion objective lens and equipped with UIS2 optical system. All images were processed using the instrument software (Olympus cellSense, Barcelona, Spain).

#### 2.3.3. ζ-potential

The ζ-potential (mV) of the oil droplets in W_1_/O/W_2_ emulsions was measured by phase-analysis light scattering (PALS) with a Zetasizer NanoZS laser diffractometer (Malvern Instruments Ltd., Worcestershire, UK). Samples were prior diluted in ultrapure water using a dilution ratio of 1:9 sample-to-solvent.

#### 2.3.4. Apparent Viscosity

Viscosity measurements (mPa·s) of the different lipid phases and W_1_/O were performed by using a vibro-viscometer (SV-10, A&D Company, Tokyo, Japan) vibrating at 30 Hz, with constant amplitude (0.4 mm) and working at 50 °C. Aliquots of 10 mL of each sample were used for determinations.

### 2.4. Colloidal Stability of W_1_/O/W_2_ Emulsions 

The stability of the prepared W_1_/O/W_2_ emulsions was measured by SLS during 12 days of dark storage at 4 °C as explained in Section 2.3.1. In addition, their stability was also performed by multiple light scattering with a Turbiscan ™Classic MA 2000 (Formulation, Toulouse, France). The turbidity measurement allows the detection of the destabilization phenomenon by multiple light scattering. Stability analysis was carried out as a variation of back scattering (BS) during storage of W_1_/O/W_2_ emulsions. The following equation was applied in order to calculate the BS:

(1) $$BS=1/\sqrt{\lambda }*$$

where λ was the photon transport mean free path in the analyzed dispersion. From the physical point of view, the λ∗(Φ,d) value in the analyzed dispersion was evaluated by using the following equation:

(2) $$\lambda *\left(\Phi ,d\right)=\;\left[\frac{2d}{3\Phi \;\left(1-g\right)Qs}\right]$$

where Φ is the volume fraction of particles, *d* is the mean diameter of particles and *g(d)* and *QS (d)* are the optical parameters given by the Mie theory [16].

Each back scattering (BS) profile obtained can be split in three zones corresponding to the bottom (on the left), the intermediate part (in the middle) and the top of the tube (on the right) where an aliquot of approximately 7 mL of the sample was placed. Emulsions destabilization mechanisms can be easily identified as BS variations in the different parts of the BS profile. Variations in particle size (flocculation or coalescence) is shown as displacement of the horizontal lines from the intermediate part of the BS profile. Whereas, gravitational separation can show up peaks either on the left part (sedimentation) or on the right (creaming). Both sedimentation and creaming can cause clarification of the emulsions. In this study, all samples showed a decrease of the BS signal on the left part of the BS profile (approx. from 0 to 50 mm), which means clarification due to gravitational movement of oil droplets to the top of the sample. Therefore, focus will be put on the analysis of BS variations on left zone of the graphs, which corresponds to the bottom of the tubes. Data analysis of the BS values are represented as BS variation (ΔBS), which refers to the BS of each storage day relative to the initial storage day.

The stability of the W_1_/O/W_2_ emulsions as determined by the BS variation was also evaluated under common storage conditions, visible light (λ = 350–700 nm) and two different storage temperatures (25 and 35 °C).

### 2.5. Encapsulation Efficiency of CHL in W_1_/O/W_2_ Emulsions

The percentage of CHL entrapped in the inner aqueous phase over 12 days of refrigerated storage (4 °C) was determined according to the methods described by Aditya et al. and Teixé-Roig et al. [2,17] with modifications. Briefly, 10 mL of the W_1_/O/W_2_ emulsion was placed in a Falcon™ tube and centrifuged (AVANTI J-25, Beckman Instruments Inc., Fullerton, CA, USA) at 4500 rpm for 10 min at 4 °C. The outer aqueous phase at the bottom of the Falcon tube (which contained the unentrapped CHL) was collected using a syringe and centrifuged at 7500 rpm for 15 min at 4 °C, prior dilution at 1:4 with methanol. The process was repeated twice in order to extract all the unentrapped CHL. Encapsulation efficiency was calculated using the following Equation (3):

(3) $$EE\left(\%\right)=\frac{N_{w1}-N_{w2}}{N_{w1}}\times 100$$

where *N_W_*_2_ is the amount of CHL seeping to the outer aqueous phase and *N_W_*_1_ is the amount of CHL added to the inner aqueous phase.

### 2.6. Statistical Analysis

All experiments were assayed in duplicate and three replicate analyses were carried out on each parameter in order to obtain mean values. An analysis of variance was carried out and the Tukey HSD test was run to determine significant differences at a 5% significance level (*p* < 0.05) with statistical software JMP Pro 14 (SAS Institute Inc., Cary, NC, USA).

## 3. Results and Discussion

### 3.1. Initial Characterization of the W_1_/O Emulsions 

Within liquid lipid phases, the fatty acid chain length of the lipid phase had a significant impact on the droplet size of the internal W_1_ droplets. CO-W_1_/O emulsions, formulated with a lipid phase with long chain fatty acids, exhibited significantly smaller average W_1_ droplet sizes in comparison with MCT-W_1_/O emulsions, which averaged 475.90 ± 63.22 and 587.47 ± 52.77 nm, respectively (Table 2). The differences in the W_1_ droplet size observed in the different lipid phases might be attributed to several reasons. On the one hand, it has been reported that the efficiency of the droplet size reduction during emulsification increases as the ratio of the dispersed phase to the continuous phase viscosities decrease [18,19]. This might be attributed to higher mechanical forces created during homogenization. Since the same aqueous phase composition (W_1_) was used for all the formulated systems, the viscosity of the lipid continuous phase might be related to the water droplet disruption efficiency. Accordingly, it was observed that CO had a higher viscosity than MCT, being 20.5 and 9.8 mPa.s, respectively. On the other hand, the oil hydrophobicity seems to also play an important role in the W_1_ droplet size of W_1_/O emulsions stabilized with PGPR. Tabibiazar & Hamishehkar observed a more compact molecular arrangement and a stronger interaction of PGPR at the water/oil interface of W_1_/O emulsions formulated with CO, which is more lipophilic, in comparison to MCT, which is less lipophilic [18]. Therefore, it is possible that the W_1_ droplet size in the present work is both dependent on the viscosity and/or the hydrophobicity of the oil used as continuous phase.

Moreover, the lipid phase state, being liquid or gelled also determined the size of the W_1_ droplets. The W_1_ droplet size in W_1_/O emulsions with a gelled lipid phase was smaller than in the respective emulsions with liquid lipid phases. W_1_ droplets with a gelled lipid phase formulated with MCT+GS presented significantly smaller W_1_ droplets, with values of 447.10 ± 120.80 nm, while in those formulated with MCT were 587.47 ± 52.77 nm (Table 2). This might be attributed to a number of reasons. On the one hand, the addition of GS in the lipid phase significantly increases its viscosity (Table 2), which might increase the emulsification efficiency due to an increase of the mechanical forces during emulsification. On the other hand, GS is a monoglyceride with interfacial activity that might present adsorption at the surface of the W_1_ droplets, thus contributing to a certain extent to the droplet size reduction during emulsification [20]. Nonetheless, this effect was less pronounced in the case of the lipid phases containing CO, with and without GS. These results can be explained by the stronger interaction of CO with the surfactant to stabilize the inner W_1_ droplets, which might favor the total covering of the water/oil interface by PGPR. As a result, GS would remain in the bulk lipid phase forming a network of crystals rather than to adsorb at the water/oil interface [21]. In fact, this hypothesis is supported by the results reported by Weiss & Muschiolik, who observed differences in the interfacial tension of MCT/fat–crystallized W_1_/O emulsions depending on the interaction between the lipid components and PGPR [19].

In concordance with the droplet size results measured by DLS, it was also possible to identify homogeneous nanometric droplets in all W_1_/O emulsions in the microscopy images (Table 3). Nevertheless, due to the limit of detection of the optic microscopy, it was not possible to visually observe differences in their droplet size when varying the lipid phase formulation.

### 3.2. Formation of W_1_/O/W_2_ Emulsions 

The influence of the surfactant type (T80 or lecithin) and the state (liquid vs. gelled) of the lipid phase on the formation of W_1_/O/W_2_ emulsions will be addressed in terms of their structure as determined by optical microscopy as well as droplet size and ζ-potential.

#### 3.2.1. Optical Microscopy

The capability of forming W_1_/O/W_2_ emulsions mainly depended on the type of hydrophilic emulsifier used to disperse the W_1_/O droplets into the W_2_ phase (Table 3), being T80 capable of forming double emulsions regardless the lipid type or state, while lecithin did not form W_1_/O/W_2_ emulsions for all the lipid types. Nevertheless, gelling the lipid phase allowed the formation of W_1_/O/W_2_ emulsions when lecithin was used as surfactant, for both lipid types (MCT and CO).

In those emulsions with a liquid lipid phase, the use of T80 led to the formation of W_1_/O/W_2_ emulsions with MCT or CO, since in both cases oil droplets filled with water droplets were observed (Table 3). T80 has a high proportion of polar groups and consequently it strongly adsorbs at the oil/water interface, which explains the results obtained [11]. On the contrary, lecithin was only able to form initially stable W_1_/O/W_2_ emulsions when CO was used as a lipid phase, while single O/W emulsions were formed when using MCT as lipid phase, evidencing a clear destabilization of the dispersed inner W_1_ droplets when MCT was used. This might be related to the lower polarity of MCT in comparison to CO [22], hence MCT being less efficient than CO in preventing the water migration from the inner to the outer aqueous phase during the emulsification process. In addition to this, lecithin presents a strong amphiphilic nature, and is preferably adsorbed in highly lipophilic interfaces, such as in CO rather than in MCT. Hence, in those emulsions formulated with MCT as lipid phase, lecithin would have been preferably located in the bulk phase, causing an osmotic imbalance between the two aqueous phases, and ultimately the destabilization of the W_1_ dispersed droplets.

When GS was used to gel the lipid phase, initially stable W_1_/O/W_2_ emulsions were obtained irrespective of the hydrophilic emulsifier and oil used (Table 3). This might be attributed to a decrease of water migration between aqueous phases due to the physical barrier formed by the presence of a GS crystal network in the gelled lipid phase. These results are consistent with recent studies on the impact of the lipid phase solidification on the resistance of W_1_/O/W_2_ emulsions to osmotic stress [23,24,25]. For instance, Liu et al. reported that under an external applied osmotic gradient, W_1_/O/W_2_ emulsions containing soybean oil experimented swelling or shrinkage, whereas semi-solid hydrogenated soybean oil W_1_/O/W_2_ emulsions remained without changes and thereby retarded the leakage of the W_1_ phase components [24].

#### 3.2.2. Droplet Size

Since the objective of this work was to study the formation and stability of W_1_/O/W_2_ emulsions, in the following sections, only those formulations rendering the formation of W_1_/O/W_2_ emulsions will be discussed, being liquid lipid W_1_/O/W_2_ emulsions formulated with lipid phases consisting on MCT or CO and stabilized with T80, and CO stabilized with lecithin. W_1_/O/W_2_ emulsions with gelled liquid phases formulated with MCT or CO mixed with GS as lipid phases and stabilized with T80 or lecithin were also included.

On the one hand, the type of oil used in order to formulate W_1_/O/W_2_ emulsions had a significant impact on the droplet size of the W_1_/O droplets stabilized with T80 (Table 4), being significantly smaller when using MCT (9.90 ± 0.15 µm) as compared to CO (13.14 ± 1.51 µm).

Other authors have reported a relationship between the dispersed phase viscosity and the final emulsion droplet size, obtaining smaller droplet sizes when using a low viscosity dispersed phase [19,26]. This might be also applicable when the dispersed phase is a water-in-oil emulsion, such as the case of the present work. In this regard, the viscosity values of the W_1_/O emulsions formulated with MCT, were significantly lower (68.7 ± 1.5 mPa·s) than the ones with CO (150.3 ± 1.5 mPa·s) (Table 2). Hence, it is reasonable to assume a relationship between the dispersed phase viscosity and the oil droplet size on the formation of W_1_/O/W_2_ emulsions. On the other hand, the surfactant type (T80 or lecithin) used to stabilize the CO-W_1_/O droplets dispersed in the W_2_ phase did not cause a significant effect on their droplet size, which ranged between 13.14 and 14.54 µm (Table 4). Both emulsifiers are classified as small-molecule emulsifiers, thus occupying the same space at the oil/water interface and consequently leading to the formation of droplets with similar sizes [27].

Regarding the lipid phase state, non-significant differences in the average oil droplet size of the W_1_/O/W_2_ emulsions formulated with MCT and GS were observed in comparison to their respective liquid emulsions (Table 4). On the contrary, CO-W_1_/O/W_2_ emulsions showed a significantly smaller average oil droplet size with a gelled (7.64–9.06 µm) than with a liquid (13.14–14.54 µm) lipid phase, regardless the emulsifier used. In a previous study of O/W emulsions, it was also observed that when the lipid phase was crystallized with GS, there was a reduction in the average oil droplet size, which was attributed to the GS surface-active properties and adsorption to the oil/water interface [15].

#### 3.2.3. ζ-potential

The ζ-potential of the W_1_/O/W_2_ emulsions is detailed in Table 4. There were no significant differences between the electrical charge (ζ-potential) of the T80-stabilized W_1_/O/W_2_ emulsions formulated with MCT or CO oil, with values ranging between −24.65 and −26.92 mV. On the contrary, the emulsifier type affected the electrical charge of the oil droplets, presenting more negative values when lecithin was used as emulsifier (−70.95 ± 4.81 mV) in comparison to T80. The lower ζ-potential values of W_1_/O/W_2_ emulsions stabilized with lecithin might be due to the anionic nature of this emulsifier, which is rich in phosphate groups $\left({\mathrm{PO}}_{4}^{3-}\right)$ [28,29]. On the contrary, T80 is a non-ionic emulsifier, meaning it does not give a charge when adsorbed at the interface. However, it is known that anionic hydroxyl groups (OH^−^) present in the water or oil used to prepare the W_1_/O/W_2_ emulsion can give small negative charges [30]. On the one hand, lecithin led to less negatively charged dispersed droplets when the lipid phase was gelled in comparison to the respective liquid phase. In this regard, ζ-potential values were −70.95 ± 4.81 and −57.52 ± 7.61 mV for CO-W_1_/O/W_2_ emulsions with liquid and gelled lipid phases, respectively (Table 4). This might be attributed to the ability of GS to displace a certain amount of anionic lecithin molecules from the oil droplet surface [31], thus contributing to the overall increase in the ζ-potential values becoming less negatively charged. On the other hand, W_1_/O/W_2_ emulsions stabilized with T80, showed similar ζ-potential values when formulated with either MCT or CO, regardless the lipid state (Table 4). This might be due to the fact that T80 strongly adsorbs at the oil/water interface, which prevents its displacement by GS molecules, hence maintaining its interfacial electrostatic characteristics [32].

### 3.3. Colloidal Stability of W_1_/O/W_2_ Emulsions

The colloidal stability of W_1_/O/W_2_ emulsions was characterized in terms of optical microscopy, as well as static and multiple light scattering during 12 days of dark storage at 4 °C in order to simulate common storage conditions.

**Figure 1:**
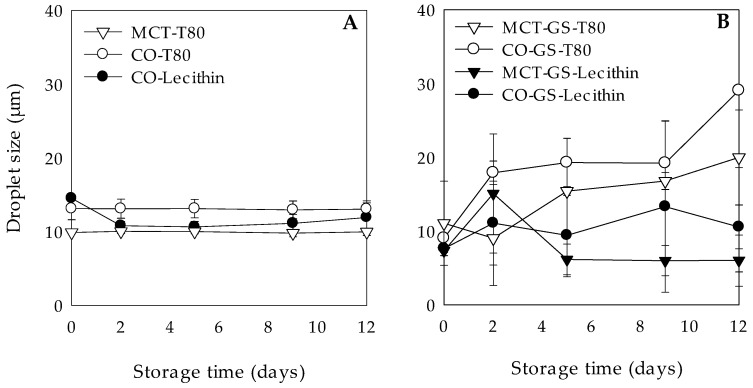
Droplet size (D_[4;3]_) during 12 days of dark storage at 4 °C of chlorophyllin-loaded W_1_/O/W_2_ emulsions formulated with different lipid phases consisting on medium chain triglyceride (MCT) or corn oil (CO) and without (**A**) or with (**B**) glyceryl stearate (GS) as well as different hydrophilic surfactants (Tween 80 (T80) or Lecithin).

As visually observed by phase contrast optical microscopy (Table 3), gelling the lipid phase with GS rendered W_1_/O/W_2_ emulsions with smaller droplet sizes after 12 days of storage regardless the type of lipid (MCT or CO) or emulsifier (T80 or lecithin) used. Nonetheless, gelling the lipid phase led to higher droplet size variations of the W_1_/O droplets dispersed in the W_2_ phase during storage time as indicated by the large droplet size deviations as measured by SLS, while the droplet size of the W_1_/O/W_2_ emulsions with liquid lipid phases remained constant with small standard deviations (Figure 1). It has been reported that O/W nanoemulsions with a liquid lipid phase have spherical shape, while solid lipid tend to be non-spherical due to the formation of crystals on the lipid phase [33]. If we consider that in the present study the droplet size was expressed as volume mean diameter, which assumes spherical droplets, a possible non-spherical shape of the W_1_/O droplets with GS may be detected as bigger oil droplets [15]. In addition, the increase on droplet size may also be attributed to oil droplets aggregation due to changes in the crystal morphology [15]. Numerous studies have observed that lipid crystals rearrange in a more stable form (from α to β) after emulsion formation [34,35,36]. As a consequence, these β-form crystals could lead to an increase in the surface-area of the oil droplets, enhancing the attraction forces between them. In the case of lecithin, droplet aggregation might have been inhibited by the high electrostatic repulsion between oil droplets (see Section 3.2.3).

**Figure 2:**
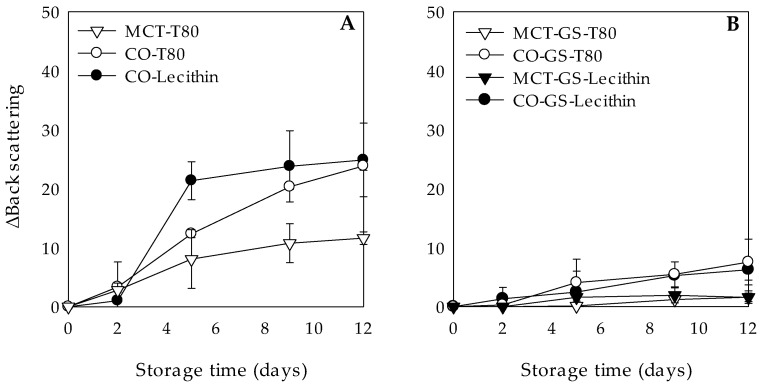
Clarification, expressed as variation of back scattering (ΔBS), at 4 °C during 12 days of dark storage of chlorophyllin-loaded W_1_/O/W_2_ emulsions formulated with different lipid phases consisting on medium chain triglyceride (MCT) or corn oil (CO) and without (**A**) or with (**B**) glyceryl stearate (GS) as well as different hydrophilic surfactants (Tween 80 (T80) or Lecithin).

Clarification, measured as ΔBS at the bottom of the tube, was also influenced by the lipid phase state. In general, W_1_/O/W_2_ emulsions with a liquid lipid phase showed a significant increase in BS values during storage time, whereas their respective W_1_/O/W_2_ emulsions with a gelled lipid phase remained without changes, which evidenced their higher stability against clarification (Figure 2). According to Fernández-Martín et al. [37], the crystallization of a lipid phase would increase its viscosity offering a higher resistance to a viscous flow. Regarding the BS changes in the W_1_/O/W_2_ emulsions with liquid lipid phases, they presented ΔBS values below 5 during the first 2 days of storage, but experimented an increase after 5 days (Figure 2A). At that point, clarification rather depended on the type of hydrophilic emulsifier used to disperse the W_1_/O droplets into the W_2_ phase, than on the lipid phase composition, with ΔBS values of around 20 and below 15 for lecithin and T80, respectively. Nevertheless, at the end of the storage time, differences in the ΔBS were predominantly due to the lipid phase composition.

For instance, W_1_/O/W_2_ emulsions stabilized with T80 presented ΔBS values of 11.64 ± 1.08 and 23.88 ± 0.74 for MCT and CO, respectively (Figure 2A). This might be due to the reduced initial oil droplet size of the W_1_/O/W_2_ emulsions formulated with MCT as compared to CO, which is known to cause droplets to be closely packed, retarding their migration to the upper part of the tube [38].

#### 3.3.1. Effect of Temperature

**Figure 3:**
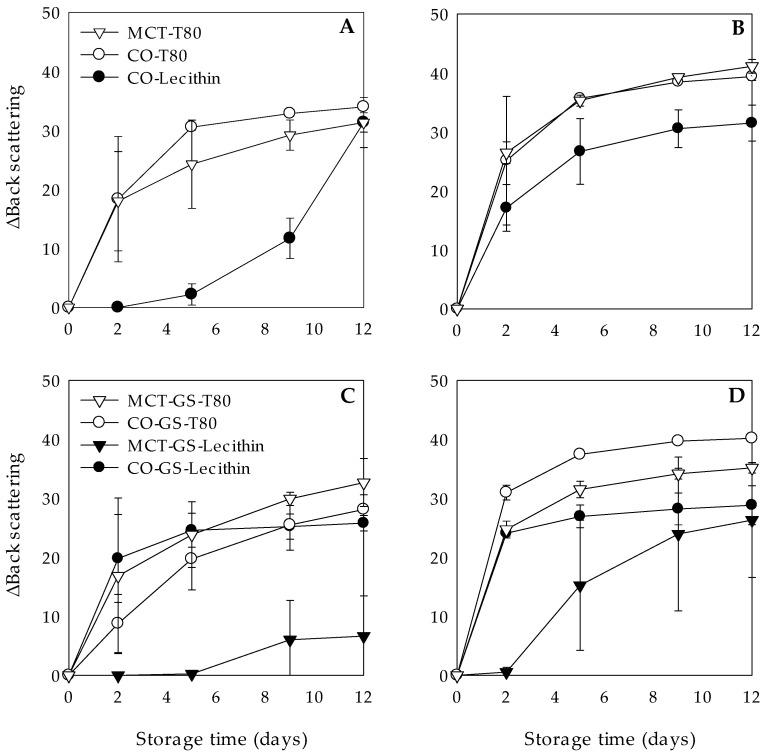
Clarification, expressed as variation of back scattering (ΔBS), at 25 °C (**A**,**C**) and 35 °C (**B**,**D**) during 12 days of dark storage of chlorophyllin-loaded W1/O/W2 emulsions formulated with different lipid phases consisting on medium chain triglyceride (MCT) or corn oil (CO) and without (**A**,**B**) or with (**C**,**D**) glyceryl stearate (GS) as well as different hydrophilic emulsifiers (Tween 80 (T80) or Lecithin).

The stability of the W_1_/O/W_2_ emulsions with liquid or gelled lipid phases stored at different temperatures (4 °C, 25 and 35 °C) against clarification is shown in Figure 3. In general, when they were stored at 25 and 35 °C, the ΔBS values obtained were higher compared to those at 4 °C, which evidenced a decrease in W_1_/O/W_2_ emulsions stability.

The extent of the liquid lipid W_1_/O/W_2_ emulsions instability stored at 25 and 35 °C was mainly dependent on the type of hydrophilic emulsifier used. After 2 days of storage, W_1_/O/W_2_ emulsions stabilized with T80 presented a noticeable increase of the ΔBS values when stored at 25 °C (ΔBS > 18) and 35 °C (ΔBS > 25) in comparison with those at 4 °C (ΔBS < 4) (Figure 2A and Figure 3A,B). This might be attributed to an increase on the free energy of the system due to the higher temperature which might result in a higher number of oil droplet collisions ultimately leading to destabilization. 

In contrast, lecithin-stabilized W_1_/O/W_2_ emulsions showed no significant differences in the ΔBS values at 4 and 25 °C, remaining stable against clarification (Figure 2A and Figure 3A). As mentioned before, lecithin-stabilized W_1_/O/W_2_ emulsions showed highly negative initial ζ-potential values (Table 4), suggesting that even at 25 °C, the electrostatic repulsion between the droplets may be high enough to inhibit droplet aggregation [39]. Nevertheless, when the storage temperature was 35 °C, an increase of ΔBS values up to 17.12 ± 3.96 was observed after 2 days of storage, suggesting that the electrostatic forces might not have been enough to overcome the attraction between the oil droplets (Figure 3B).

At the end of the storage time, all liquid lipid W_1_/O/W_2_ emulsions stored at 25 and 35 °C showed phase separation due to clarification phenomenon (Figure 3A,B).

Clarification phenomenon of the W_1_/O/W_2_ emulsions with gelled lipid phase at 25 and 35 °C was more pronounced than in their respective emulsions with liquid lipid phases. Indeed, they presented ΔBS values above 8 already from day 2 of storage at both 25 and 35 °C, regardless the type of lipid or emulsifier (Figure 3C,D). This might be attributed to changes in the lipid phase state when increasing the storage temperature from 4 to 25 and 35 °C [40,41] (Table 1). At 4 °C, gelation and/or partial crystallization of the dispersed phase significantly increases its viscosity, and consequently the stability of the W_1_/O/W_2_ emulsions (Section 3.3). At higher storage temperatures, especially at 35 °C, visual observations of the lipid phase showed a loss of structural consistency, which might be caused by its lower gelling and or crystallization degree (Table 1). As a consequence, W_1_/O/W_2_ emulsions with gelled lipid phase at 25 and 35 °C, would behave as liquid lipid emulsions, explaining the observed instabilities.

Interestingly, gelling the MCT lipid phase allowed the formation of highly stable W_1_/O/W_2_ emulsions at all the studied temperatures, in fact, they had no significant differences in the ΔBS values during the first 5 days of storage (Figure 2B and Figure 3C,D). Instead, their respective liquid lipid W_1_/O/W_2_ emulsions could not even been formed (Section 3.2.1).

#### 3.3.2. Effect of Light Exposure

**Figure 4:**
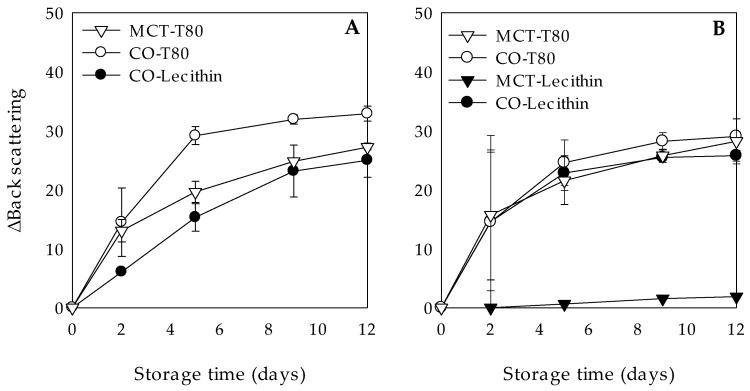
Clarification, expressed as variation of back scattering (ΔBS), at 25 °C during 12 days of light exposure of chlorophyllin-loaded W_1_/O/W_2_ emulsions formulated with different lipid phases consisting on medium chain triglyceride (MCT) or corn oil (CO) and without (**A**) or with (**B**) glyceryl stearate (GS) as well as different hydrophilic surfactants (Tween 80 (T80) or Lecithin).

W_1_/O/W_2_ emulsions stability against clarification was also evaluated when subjected to light exposure during 12 days of storage at 25 °C (Figure 4). At the end of the storage time, all W_1_/O/W_2_ emulsions (gelled and liquid lipid phase) showed no significant changes on the ΔBS values when exposed to light (Figure 4A,B) as compared to those stored in the dark (Figure 3A,C). Based on a previous research, where the effect of light exposure on the stability of emulsions with a lipid solidified phase was studied, it would have been expected to observe an increase in clarification due to an accelerated droplet growth [42]. These authors reported that high energetic radiations caused droplet collisions, leading to droplet aggregation and destabilization of the emulsion systems. However, in the present study a low intensity light was used, which has been reported to have no negative effects on emulsion stability [43].

### 3.4. Encapsulation Efficiency of CHL in W_1_/O/W_2_ Emulsions

**Figure 5:**
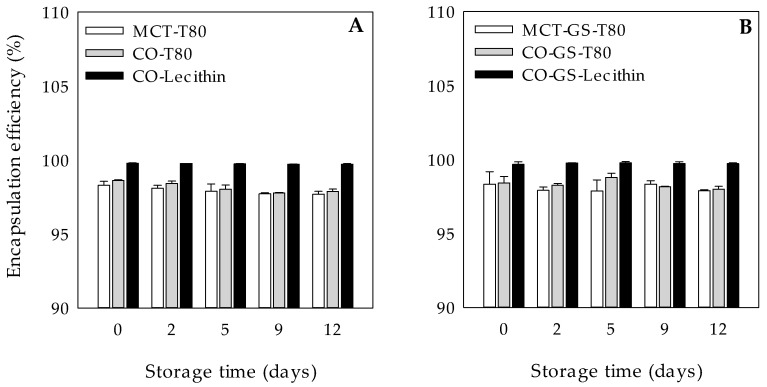
Encapsulation efficiency (%) during 12 days of dark storage at 4 °C of chlororphyllin-loaded W_1_/O/W_2_ emulsions formulated with different lipid phases consisting on medium chain triglyceride (MCT) or corn oil (CO) and without (**A**) or with (**B**) glyceryl stearate (GS) as well as different hydrophilic surfactants (Tween 80 (T80) or Lecithin).

Finally, the ability of the fresh W_1_/O/W_2_ emulsions to encapsulate CHL in the inner aqueous phase was evaluated (Figure 5). W_1_/O/W_2_ emulsions both with gelled and liquid lipid phases showed CHL EE values higher than 98%. Our results are in agreement with a previous study where the CHL EE values in liquid lipid W_1_/O/W_2_ emulsions were around 91% [3]. Interestingly, in this study, emulsions containing lecithin had the highest CHL EE, which might be due to a possible interaction between the phosphate ions of lecithin and the hydroxyl groups of the encapsulated compound, being capable of forming H-bonds with the CHL. It is worth mentioning that this is the first study in which W_1_/O/W_2_ emulsions with a gelled lipid phase are used for CHL encapsulation.

## 4. Conclusions

The present work evidences that the formation and stabilization of double W_1_/O/W_2_ emulsions can be enhanced with the use of gelled lipid phases. When liquid phases were used, only T80 was able to form W_1_/O/W_2_ emulsions both with CO or MCT liquid oils, while lecithin only rendered double emulsions with CO. With gelled lipid phases containing 1% (*w*/*w*) GS, W_1_/O/W_2_ emulsions were successfully formed regardless the lipid and surfactant type, showing also smaller inner water droplets (W_1_) and smaller lipid (W_1_/O) droplets in comparison to the respective formulations with liquid oils. This may be attributed to a decrease in the migration of water from the inner to the outer aqueous phase by the gelled lipid phase. Additionally, it was evidenced that their long-term stability under different storage temperatures was dependent on the lipid phase state. At 4 °C, the gelled and/or crystallized lipid phase contributed to a higher W_1_/O/W_2_ emulsions stability in comparison to liquid lipid emulsions. Hence this work contributes in elucidating the role of the lipid phase state, being liquid or gelled, on the formation and stabilization of W_1_/O/W_2_ emulsions that may act as carriers of hydrophilic bioactive compounds.

## Figures and Tables

**Table 1:** Visual appearance at 4, 25 and 35 °C of the gelled lipid phases consisting on blends of medium chain triglyceride (MCT) or corn oil (CO), GS (1% *w*/*w*) and PGPR.

Lipid Type	4 °C	25 °C	35 °C
MCT	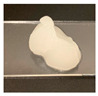	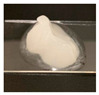	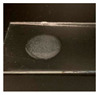
CO	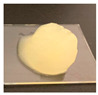	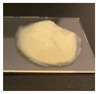	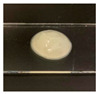

**Table 2:** Droplet size (nm) and apparent viscosity (mPa·s) of chlorophyllin-loaded W_1_/O emulsions formulated with different lipid phases consisting on medium chain triglyceride (MCT) or corn oil (CO) and without or with glyceryl stearate (GS). Different upper case letters (A, B) indicate significant differences between lipid type. Different lower case letters (a, b) indicate significant differences between different lipid states.

W_1_/O Emulsions			
	Lipid Type	Droplet Size (nm)	Apparent Viscosity (mPa·s) ^1^
Liquid lipids	MCT	587.47 ± 52.77 ^A,a^	68.7 ± 1.5 ^A,a^
	CO	475.90 ± 63.22 ^B,a^	150.3 ± 1.5 ^B,a^
Solid lipids	MCT-GS	447.10 ± 120.80 ^A,b^	116.0 ± 2.6 ^A,b^
	CO-GS	433.53 ± 235.80 ^A,a^	308.5 ± 14.4 ^B,b^

^1^ Apparent viscosity values were mesured at 50 ± 1 °C.

**Table 3:** Optical microscopy images of chlorophyllin-loaded W_1_/O emulsions (day 0) and W_1_/O/W_2_ (day 0 and 12) formulated with different lipid phases consisting on medium chain triglyceride (MCT) or corn oil (CO) and without or with glyceryl stearate (GS) as well as different hydrophilic surfactants (Tween 80 (T80) or Lecithin). Scale bar: 10 µm.

Lipid Type	W_1_/O Emulsions	W_1_/O/W_2_ Emulsions—Day 0		W_1_/O/W_2_ Emulsions—Day 12	
	PGPR	T80	Lecithin	T80	Lecithin
MCT	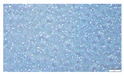	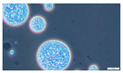	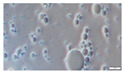	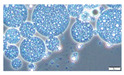	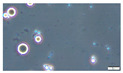
CO	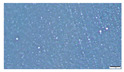	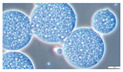	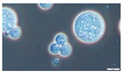	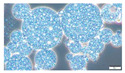	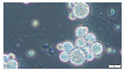
MCT-GS	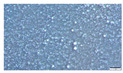	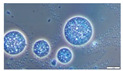	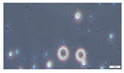	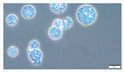	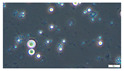
CO-GS	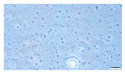	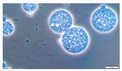	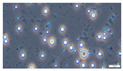	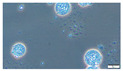	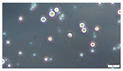

**Table 4:** Droplet size (D_[4;3]_) and ζ-potential (mV) of chlorophyllin-loaded W_1_/O/W_2_ emulsions formulated with different lipid phases consisting on medium chain triglyceride (MCT) or corn oil (CO) and without or with glyceryl stearate (GS) as well as different hydrophilic surfactants (Tween 80 (T80) or Lecithin). A, B indicates significant differences between the lipid type. a, b indicates significant differences between the used surfactants. x, y indicates significant differences between the lipid phase state.

W_1_/O/W_2_ Emulsions			
	Lipid-Emulsifier Type	D_[4;3]_ (µm)	ζ-Potential (mV)
Liquid lipids	MCT-T80	9.90 ± 0.15 ^A,a,x^	−24.65 ± 3.44 ^A,a,x^
	CO-T80	13.14 ± 1.51 ^B,a,x^	−26.92 ± 5.02 ^A,a,x^
	CO-Lecithin	14.54 ±0.14 ^B,a,x^	−70.95 ± 4.81 ^B,b,x^
Solid lipids	MCT-GS-T80	11.09 ± 5.71 ^A,a,x^	−25.06 ± 1.64 ^A,a,x^
	CO-GS-T80	9.06 ± 1.96 ^A,a,y^	−30.01 ± 5.72 ^B,a,x^
	MCT-GS-Lecithin	7.35 ± 0.68 ^A,b,y^	−63.52 ± 2.90 ^A,b,y^
	CO-GS-Lecithin	7.64 ± 0.45 ^A,b,y^	−57.52 ± 7.61 ^B,b,y^

## Data Availability

Data sharing not applicable.

## Sample Availability

Samples of the compounds are not available from the authors.
